# The nature of free-carrier transport in organometal halide perovskites

**DOI:** 10.1038/srep19599

**Published:** 2016-01-19

**Authors:** Tomoya Hakamata, Kohei Shimamura, Fuyuki Shimojo, Rajiv K. Kalia, Aiichiro Nakano, Priya Vashishta

**Affiliations:** 1 Department of Physics, Kumamoto University, Kumamoto 860-8555, Japan; 2 Collaboratory for Advanced Computing and Simulations, Department of Physics & Astronomy, Department of Computer Science, Department of Chemical Engineering & Materials Science, and Department of Biological Sciences, University of Southern California, Los Angeles, CA 90089-0242, USA; 3 Graduate School of System Informatics, Kobe University, Kobe 657-8501, Japan

## Abstract

Organometal halide perovskites are attracting great attention as promising material for solar cells because of their high power conversion efficiency. The high performance has been attributed to the existence of free charge carriers and their large diffusion lengths, but the nature of carrier transport at the atomistic level remains elusive. Here, nonadiabatic quantum molecular dynamics simulations elucidate the mechanisms underlying the excellent free-carrier transport in CH_3_NH_3_PbI_3_. Pb and I sublattices act as disjunct pathways for rapid and balanced transport of photoexcited electrons and holes, respectively, while minimizing efficiency-degrading charge recombination. On the other hand, CH_3_NH_3_ sublattice quickly screens out electrostatic electron-hole attraction to generate free carriers within 1 ps. Together this nano-architecture lets photoexcited electrons and holes dissociate instantaneously and travel far away to be harvested before dissipated as heat. This work provides much needed structure-property relationships and time-resolved information that potentially lead to rational design of efficient solar cells.

Organometal halide perovskites such as methylammonium lead iodide (CH_3_NH_3_PbI_3_ or MAPbI_3_)^1^ are attracting a great deal of attention as promising material for solar cells because of their high power conversion efficiency^2^,^3^,^4^,^5^,^6^ exceeding 20%^7^,^8^. The high performance has generally been attributed to excellent transport properties of photoexcited charge carriers^7^,^8^. Absorption of photons creates pairs of electrons and holes. A photoexcited electron-hole pair is either bound into an exciton via electrostatic attraction, or the electron and hole act as separate free carriers of negative and positive charges, respectively. Recent experiments have provided clear evidence for the existence of free charge carriers as opposed to bound excitons in MAPbI_3_^9^,^10^,^11^. Furthermore, exceptionally large diffusion lengths (>1 μm) have been observed for these free carriers^12^,^13^. As a result, a large fraction of photoexcited charges can be collected as electric current without being dissipated as heat, thereby leading to high power-conversion efficiency. Balanced diffusion lengths observed between electrons and holes make photon-to-electric current conversion even more efficient^14^.

The nature of free-carrier transport in MAPbI_3_ has not been elucidated at the atomistic level. The key questions are: Why do free charge carriers exist, and what mechanisms make their diffusion lengths so large? If we can identify key structural features for the excellent transport properties of MAPbI_3_, we may be able to utilize the structure-property relationships for rationally designing even more efficient solar cells. To answer these fundamental questions, we performed quantum molecular dynamics (QMD) and nonadiabatic quantum molecular dynamics (NAQMD) simulations of photoexcited carrier dynamics in MAPbI_3_; see supplementary information for simulation details. QMD follows the trajectories of all atoms, while computing interatomic forces quantum mechanically from first principles^15^. NAQMD describes electronic excitations and nonadiabatic transitions between excited electronic states assisted by atomic motions, thereby describing photoexcitation dynamics involving electrons and nuclei^16^.

We performed two sets of simulations involving 2 × 2 × 2 and 3 × 3 × 3 MAPbI_3_ crystalline unit cells, respectively (Fig. 1a). Each cubic unit cell contains one Pb atom at the body center and I atoms at the face centers to form a PbI_6_ octahedron, whereas MA molecules at the corners of the cube rotate rather freely. The simulations were performed at a temperature of 300 K. Figure 1b shows the time evolution of electronic Kohn-Sham (KS) eigenenergies near the bottom of the unoccupied conduction band (CB) around 1 eV and the top of the occupied valence band (VB) around −1 eV in the 2 × 2 × 2 unit-cell simulation. Here, the origin of the energy is Fermi energy. We observe a large number of electronic level crossings assisted by thermal motion of atoms. This demonstrates highly degenerate energy levels at the CB bottom and VB top.

Next, we studied the character of wave functions as well as their time evolution. We projected the electronic wave functions at the CB-bottom and VB-top onto pseudoatomic orbitals of different angular momenta (*i.e*., s, p, and d) centered around different atoms^17^. Figure 1c shows the time evolution of the composition of the CB-bottom and VB-top wave functions during QMD simulation. An important observation is that these frontier orbitals reside exclusively in the Pb-I sublattice but not on MA molecules, which is in agreement with previous theoretical calculation^18^. Time-averaged composition shows that the CB-bottom wave function comes from 6p state of Pb (63%), 5d state of I (27%), and 5p state of I (10%). On the other hand, the VB-top wave function comes mostly from 5p state of I (90%) with a slight mixture (10%) of Pb-6s state. The dominance of Pb-6p and I-5p states in CB-bottom and VB-top wave functions is in agreement with previous theoretical works^19^. In addition, our analysis shows significant involvement of I-5d state, and provides quantitative information on compositions that will be critical for discussing carrier transport mechanisms below. Figure 1d shows partial electronic density of states (DOS) projected onto atomic species. Consistently with the above analysis, the DOS of CB at positive energy and that of VB at negative energy are dominated by Pb and I contributions, respectively.

To study photoexcited charge-carrier dynamics, we next performed NAQMD simulations. The simulations were initiated by exciting the VB-top electron to the empty CB-bottom state. For each of the 2 × 2 × 2 and 3 × 3 × 3 unit-cell systems, three NAQMD simulations were performed at 300 K starting with different initial atomic configurations sampled from the QMD simulations discussed above. We first studied the nature of charge carriers by calculating the exciton binding energy between the photoexcited electron and hole. To do so, we estimated the electronic excitation energy *E*_exc_ including many-body effects in the framework of linear-response time-dependent density functional theory (LR-TDDFT). Our LR-TDDFT calculation incorporates long-range exact exchange correction, which was shown to describe excitonic binding adequately^20^,^21^. In the calculation, each electronic excited state was represented by a linear combination of 10 Slater determinants. The exciton binding energy was then estimated by subtracting the energy difference between the dominant electron and hole states, Δ*ε*_e−h_ = *ε*_electron_ − *ε*_hole_, from *E*_exc_. Figure 2a compares the time evolution of *E*_exc_ (red) and Δ*ε*_e−h_ (black). The two curves are nearly identical, demonstrating very weak exciton binding. The time averaged exciton binding energy is 〈*E*_exc_ – Δ*ε*_e−h_〉 = −0.012 ± 0.009 eV. Such a weakly bound exciton can easily dissociate by thermal energy, 0.026 eV, at room temperature, thereby generating an electron and a hole that freely move independently of each other. Our *ab initio* exciton binding energy (0.012 eV) is below an experimentally derived upper bound (0.05 eV)^22^, and is consistent with recent conjectures (≤0.01 eV)^8^. The weak exciton binding in MAPbI_3_ is in sharp contrast to strong exciton binding in most organic solar cells^23^. Our NAQMD simulation thus demonstrates that photoexcited charge carriers in MAPbI_3_ are free electrons and holes instead of strongly bound excitons in conventional organic photovoltaic materials. This result is consistent with experimentally inferred existence of free charge carriers in organometal halide perovskites^9^,^11^.

Figure 2b shows the time evolution of KS eigenenergies in the NAQMD simulation, where red and blue curves are energies of KS states that mainly contribute to quasielectron and quasihole, respectively. It should be noted that KS states here are distinct from those in QMD simulations due to the use of interatomic forces that reflect electronic excitations. We observe a large number of electronic level crossings assisted by thermal motion of atoms. Accordingly, frequent electronic transitions occur between excited electron and hole states.

Supplementary Fig. S1 shows the time evolution of the composition of photoexcited quasielectron and quasihole charge densities. The characters of photoexcited electron and hole charge densities during the NAQMD simulation in Fig. S1 are nearly identical to those of CB-bottom and VB-top wave functions during the QMD simulation in Fig. 1c. Namely, the photoexcited electron is composed of Pb-6p states, whereas the hole is mostly composed of I-5p state. Thus, photoexcited electrons predominantly travel on a sublattice composed of Pb atoms, whereas holes travel independently on a disjoint sublattice composed of I atoms.

To quantify the spatial extent of free carriers, we calculated the participation number^23^, 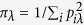 (*λ* = e or h for electron or hole, respectively). Here, 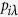 is the existing probability of the quasielectron or quasihole on the *i*^th^ atom calculated by Mulliken analysis^23^. The 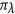 value reflects the number of atoms over which the carrier is spread. Figure 2c shows 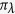 as a function of time in a NAQMD simulation of 2 × 2 × 2 unit cells. 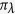 switches between large (~15) and small (~3) values. Comparison of Fig. 2c with Fig. 2b shows that the participation number becomes large when energy levels are degenerate. An interesting observation is the slight anti-correlation of *π*_e_ and *π*_h_ values (Pearson correlation coefficient of –0.029), *i.e*., an electron is slightly extended when a hole is localized, and vice versa.

To quantify the free-carrier transport, we estimated the diffusion coefficients of electrons and holes. At each NAQMD step, center-of-mass (COM) positions for the electron and hole were determined from the existing probability 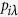 of the electron and hole on the *i*^th^ atom^23^, and diffusion coefficients were calculated from the slopes of the mean square displacements (MSD) as a function of time. Figure 2d shows the electron and hole MSD as a function of time in a 2 × 2 × 2 unit-cell NAQMD simulation. Here, sampling was performed over 30 time origins within each NAQMD trajectory. From the linear regression, we obtained the electron and hole diffusion coefficient to be *D*_e_ = (1.16 ± 0.31) × 10^−2^ cm^2^/s and *D*_h_ = (1.01 ± 0.42) × 10^−2^ cm^2^/s. The NAQMD simulation results are in good agreement with recent experimental data: *D*_*e*_ = (1.7 ± 1.1) × 10^−2^ cm^2^/s and *D*_h_ = (1.1 ± 0.7) × 10^−2^ cm^2^/s^12^. As shown in supplementary information, finite-size effects on the COM calculation are negligible (Fig. S2). This analysis of finite-size effects indicates that electron and hole wave functions are not percolating through the system and act as well defined wave packets, likely due to thermal disorder. It should be noted that the thermal disorder-induced carrier (*i.e*., electron and hole) localization does not contradict with the experimentally inferred large exciton sizes in MAPbI_3_. For example, magnetoabsorption measurements were used to infer the exciton size to be 2.2 nm^24^. However, this large exciton size simply reflects the weak binding between an electron and a hole, which easily dissociate at room temperature as explained earlier. Thus the exciton size here is not a physically important quantity. Once dissociated, the electron and hole instead act as free carriers that diffuse rapidly while being localized individually by thermal disorder.

We next calculated the radiative recombination time *τ* of photoexcited carriers. Figure 3a shows the calculated 1/*τ* as a function of time in a 3 × 3 × 3 unit-cell NAQMD simulation. We observe peaks, which coincide with the times when bands are not degenerate (*i.e*., away from level crossings). The average recombination time is *τ* = 0.87 ± 0.53 ns in the 2 × 2 × 2 simulations, where the carrier density is *ρ* = 4.77 × 10^20^ cm^−3^ and *τ* = 3.88 ± 1.93 ns in the 3 × 3 × 3 simulations, where the carrier density is *ρ* = 1.47×10^20^ cm^−3^. The decrease in the recombination time as a function of *ρ* is consistent with the dominance of free carriers^9^,^11^.

From the calculated diffusion coefficient and recombination time for photoexcited electrons and holes, we calculated the diffusion lengths 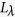
= (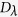
*τ*)^1/2^ (*λ* = e or h for electron or hole, respectively). Even for a very high charge carrier density of *ρ* = 4.77 × 10^20^ cm^−3^, our NAQMD results, *L*_e_ = 31.7 ± 25.9 nm and *L*_h_ = 29.5 ± 25.5 nm, are rather large. The calculated large diffusion lengths may be understood as follows. As described earlier, Pb and I sublattices act as separate pathways for rapid transport of electrons and holes, respectively. Since electrons and holes move on spatially distinct pathways, the overlap of their wave functions is small, thereby reducing their radiative recombination rate.

To understand microscopic mechanisms underlying the large free-carrier diffusion lengths, we analyzed the dynamics of electron and hole transport. Figure 3b and supplementary movie, S1.mov, show quasielectron (red) and quasihole (blue) charge densities in a 3 × 3 × 3 unit-cell NAQMD simulation. Supplementary movie, S2.mov, animates the quasielectron and quasihole COM positions as well as the atoms occupied by electron and hole wave functions. We observe that participation number *π*_e_ is small when the hole is degenerate. When the wave function of a carrier is localized, it cannot diffuse. On the other hand, when the wave function is spread out, the carrier diffuses rapidly. Most importantly, the calculated diffusion lengths for electrons and holes are balanced, which is consistent with recent experimental observations^14^. The balanced diffusion lengths of electrons and holes are important for photoexcited charge carriers to be collected as electric current, thus leading to high power-conversion efficiency. The balanced electron and hole diffusion may be understood as follows. Since electrons and holes reside on Pb and I atoms, their COM positions move along Pb and I sublattices, respectively, as shown in S2.mov. The distance between nearest-neighbor Pb atoms is the lattice constant *a* ~ 6.33 Å, while that of neighbor I atoms is *a*/√2 ~ 4.48 Å, *i.e*., holes in I sublattice need to make more hops than electrons in Pb sublattice to travel the same distance. To quantify the spread of electrons and holes on these sublattices, we define partial participation number 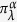 by counting only Pb atoms for electrons and only I atoms for holes, respectively (*α* = Pb or I for electron or hole, respectively). The time-averaged 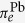 and 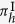 are 3.3 ± 1.3 and 8.2 ± 3.1, respectively, *i.e.*, each electron or hole resides on approximately ~3 Pb or ~8 I atoms. This trade-off between wave-function localization and elementary hopping distance partly explains why experimentally observed electron and hole diffusion constants are balanced^14^ despite their different effective masses^25^.

A key characteristic of organometal halide perovskites is the large electric polarizability of the organometal sublattice. The organometal sublattice is composed of methylammonium molecules that have intramolecular dipoles. These dipoles can freely rotate and screen the Coulombic interaction between photoexcited electrons and holes in the Pb and I sublattices. To quantify the electric polarizability, we computed the dielectric constant using the fluctuation-dissipation theorem (see supplementary information). The dielectric constant *ε* calculated for the QMD simulations is ~4 (Fig. 4a). Considering that our QMD simulation for 10^−12^ s only samples dipole fluctuations of frequency above 10^12^ Hz, the calculated *ε* is in reasonable agreement with experimental values of 7–10 at 10^12^ Hz^8^. On the other hand, the result for the NAQMD simulations in Fig. 4b is rather large. As seen in S1.mov, the large polarizability of MA sublattice likely arises from free rotations of MA molecules. MA sublattice effectively screens out electrostatic electron-hole attraction to unbind excitons, thereby generating free electrons and holes. Figure 4c,d, shows the autocorrelation function of the dipole moment **M**,


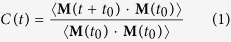


for the QMD and NAQMD simulations, respectively, where 〈〉 denotes averaging over the time origin *t*_0_. The correlation time of dipole moments, estimated from the decay of *C*(*t*), is ~0.49 ps for the QMD simulations (Fig. 4c) and ~0.25 ps for the NAQMD simulations (Fig. 4d). These correlation times correspond to experimental estimates of the C-N rotation time, 0.46 ps, in MAPbI_3_^26^. This rapid dielectric response dissociates an exciton within ~1 ps. This is consistent with the experimentally inferred exciton dissociation time of ~2 ps^8^. We found major contributions of dipole moments of hydrogen bonds between H atoms in MA molecules and I atoms to *ε*. I atoms form massive hydrogen bonds with H atoms in the nearest neighbor MA molecules as can be seen in Fig. 1a. Charge fluctuations due to continuous formation and breakage of these hydrogen bonds make large contribution to *ε*. This is also consistent with the C-N rotation time and the correlation time of dipole-moment dynamics.

The essence of efficient solar energy utilization are to: (i) separate a photoexcited electron-hole pair into an electron and a hole as quickly as possible; and (ii) let the free electron and hole travel as far away from each other as possible until they are harvested separately before being dissipated as heat. The above first principles results clearly identify the key nano-structural and dynamic features that satisfy both requirements in high-performance organometal halide solar cells: Fast-polarizing organic sublattice for (i); and separate metal and halide express lanes for electrons and holes, respectively, for (ii).

## Methods

We simulated two system sizes for MAPbI_3_ crystal: (i) 2 × 2 × 2 crystalline unit cells containing 96 atoms in a cubic simulation box of side 12.66 Å; and (ii) 3 × 3 × 3 crystalline unit cells containing 324 atoms in a cubic simulation box of side 18.99 Å. Periodic boundary conditions were applied in all three Cartesian directions, and the Γ point was used for Brillouin-zone sampling. We performed simulations at temperature 300 K in the canonical ensemble. The equations of motion were integrated numerically with a time step of 0.484 fs. QMD simulations were performed for the time duration of 2.904 ps for the 2 × 2 × 2 and 3 × 3 × 3 MAPbI_3_ crystalline unit cells. Subsequently, we performed three NAQMD simulations for each system size, starting with different atomic configurations sampled from the QMD simulations. Simulated times in the NAQMD simulations were 4.356, 2.420 and 2.178 ps for 2 × 2 × 2 MAPbI_3_ crystalline unit cells, and 0.944, 0.242 and 1.089 ps for 3 × 3 × 3 unit cells. The full detail of simulation methods is included in supplementary information.

## Additional Information

**How to cite this article**: Hakamata, T. *et al*. The nature of free-carrier transport in organometal halide perovskites *Sci. Rep.* **6**, 19599; doi: 10.1038/srep19599 (2016).

## Supplementary Material

Supplementary Information

Supplementary Movie S1

Supplementary Movie S2

## Figures and Tables

**Figure 1:**
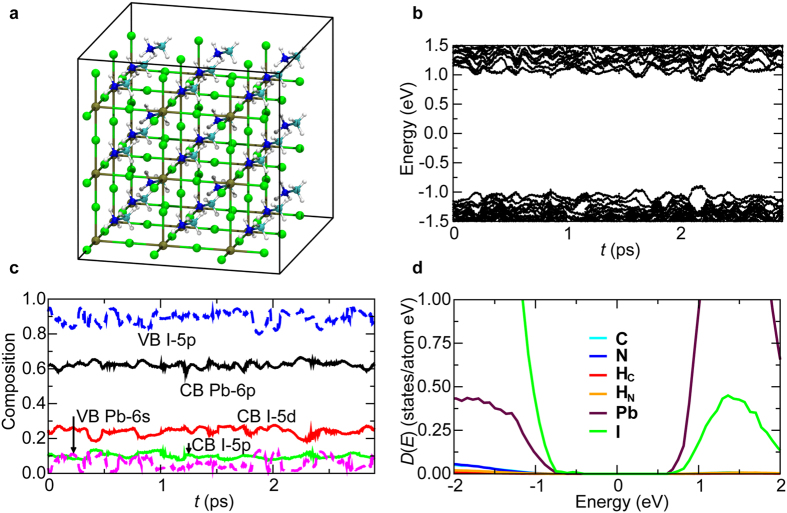
Electronic wave functions in MAPbI_3_. (**a**) Simulated MAPbI_3_ crystal with 3 × 3 × 3 unit cells, where H, C, N, I, and Pb atoms are colored in white, cyan, blue, green, and brown, respectively. (**b**) Time evolution of KS eigenenergies near the Fermi energy in the 2 × 2 × 2 unit-cell QMD simulation. (**c**) Time evolution of the projection of CB-bottom and VB-top wave functions onto different angular momenta (*i.e*., s, p, and d) around different atoms. (**d**) Partial electronic DOS.

**Figure 2:**
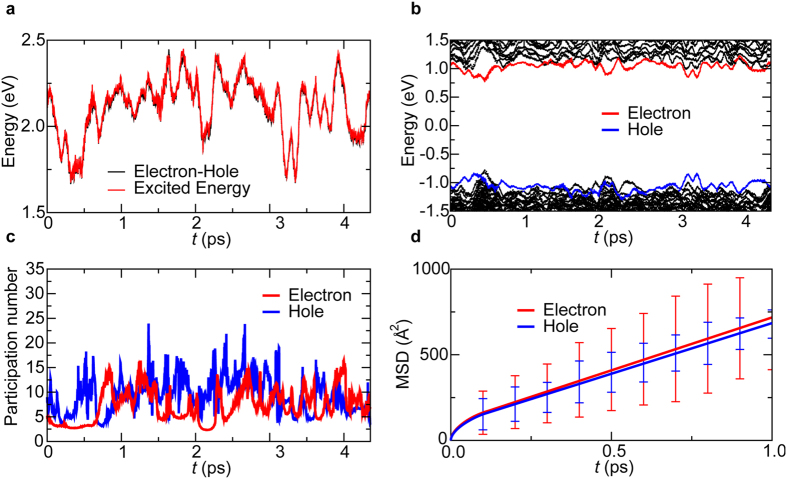
Rapid and balanced transport of photoexcited electrons and holes. (**a**) Time evolution of the many-body electronic excitation energy *E*_exc_ including excitonic binding (red) compared with the energy difference between the electron and hole, Δ*ε*_e−h_ = *ε*_electron_ − *ε*_hole_ (black) in a 2 × 2 × 2 unit-cell NAQMD simulation. **(b**) Time evolution of KS eigenenergies, where the KS states that contribute mainly to quasielectron and quasihole are colored red and blue, respectively. (**c**) Electron (red) and hole (blue) participation numbers as a function of time. (**d**) MSD of photoexcited electrons and holes as a function of time.

**Figure 3:**
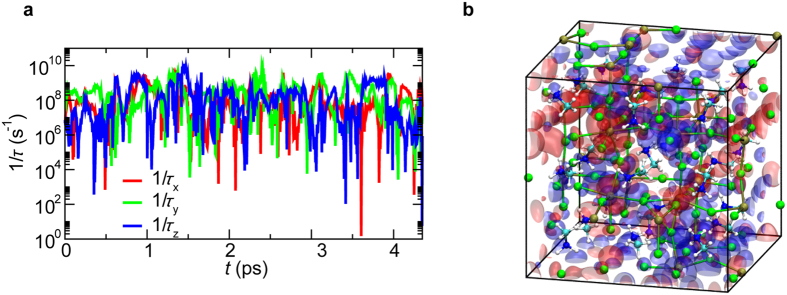
Limited recombination of electrons and holes. (**a)** Inverse radiative recombination time (1/*τ*_*x*_, 1/*τ*_*y*_, 1/*τ*_*z*_) for polarization along the *x, y* and *z* axes as a function of time in a 3 × 3 × 3 unit-cell NAQMD simulation. (**b**) A snapshot of quasielectron (red) and quasihole (blue) charge densities, where contour surfaces of 1 × 10^−4^ a.u.^−3^ are shown.

**Figure 4:**
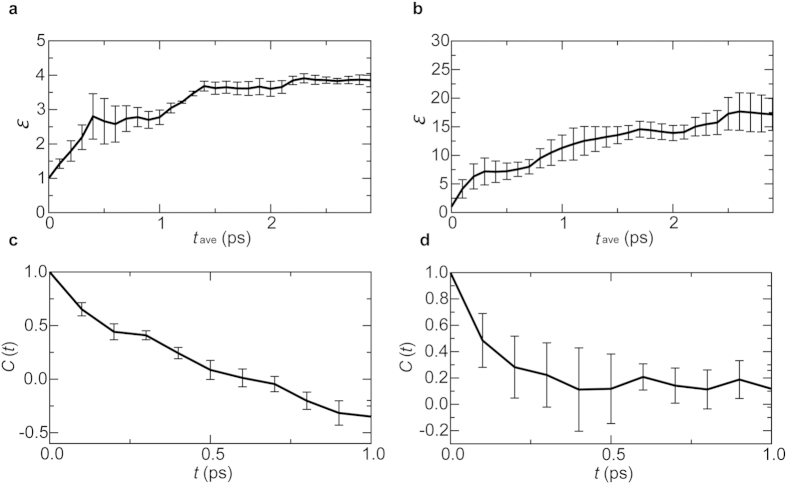
Charge-carrier screening. (**a**) Dielectric constant in the QMD simulations as a function of the extent of time averaging. (**b**) Dielectric constant in the NAQMD simulations as a function of the extent of time averaging. (**c**) Autocorrelation function of electric dipole moment in the QMD simulations. (**d**) Autocorrelation function of electric dipole moment in the NAQMD simulations.
