# Supplementary material for: The nature of free-carrier transport in organometal halide perovskites
Source: Sci Rep. 2016 Jan 19;6:19599. doi: 10.1038/srep19599 (PMC4726051; doi:10.1038/srep19599)
Supplement: Supplementary Information [file srep19599-s1.pdf]

SUPPLEMENTARY INFORMATION

The nature of free-carrier transport in organometal halide perovskites

Tomoya Hakamata,[1] Kohei Shimamura,[1,2,3] Fuyuki Shimojo,[1] Rajiv K. Kalia,[2]
Aiichiro Nakano,[2*] and Priya Vashishta[2]

[1]Department of Physics, Kumamoto University, Kumamoto 860-8555, Japan.
[2]Collaboratory for Advanced Computing and Simulations, Department of Physics & Astronomy, Department of Computer Science, Department of Chemical Engineering & Materials Science, and Department of Biological Sciences, University of Southern California, Los Angeles, CA 90089-0242, USA.
[3]Graduate School of System Informatics, Kobe University, Kobe 657-8501, Japan.
*Corresponding author. Email: anakano@usc.edu

Overview.

Simulation methods

Description of movies S1.mov and S2.mov

Figures S1 and S2

Simulation Methods.

Quantum molecular dynamics (QMD) and nonadiabatic quantum molecular dynamics (NAQMD) simulation methods: The electronic states were calculated using the projector-augmented-wave (PAW) method[1], which is an all-electron electronic-structure-calculation method within the frozen-core approximation. Projector functions were generated for 6s, 6p and 6d states of Pb, 5s, 5p and 5d states of I, 2s and 2p states of C and O, and 1s state of H. In the framework of density functional theory (DFT)[2], the generalized gradient approximation (GGA)[3] was used for the exchange-correlation energy with nonlinear core corrections[4]. The momentum-space formalism was utilized[5], where the plane-wave cutoff energies were 25 and 250 Ry for electronic pseudo-wave functions and pseudo-charge density, respectively. The energy functional was minimized iteratively using a preconditioned conjugate-gradient method[6,7]. QMD simulations were carried out using quantum mechanically computed interatomic forces based on the Hellmann-Feynman theorem[8]. We used our own QMD code[9,10]. The code has been implemented on parallel computers[7] by a hybrid approach combining spatial decomposition (*i.e.*, distributing real-space or reciprocal-space grid points among processors) and band decomposition (*i.e.*, assigning the calculations of different electronic wave functions to different processors). The program is written using the message passing interface (MPI) library for interprocessor communications.

To study the dynamics of photoexcited charge carriers, we performed NAQMD simulations[11-15]. The NAQMD method describes electronic excitations in the framework of linear-response time-dependent density functional theory[16]. In addition, nonadiabatic transitions between excited electronic states assisted by molecular motions are treated with a surface-hopping approach[17-21]. To perform large NAQMD simulations involving many hundreds of

atoms, we have implemented a series of techniques for efficiently calculating long-range exact exchange correction and excited-state forces[22,23]. Due to the use of excited-state forces, photoexcitation also modifies ground-state electronic structures. Details of our QMD and NAQMD simulation software are described in Ref. 23.

Bond-overlap population analysis: To quantify the change in the bonding properties of atoms, we used bond-overlap population analysis[24] by expanding the electronic wave functions in an atomic-orbital basis set[25]. Based on the formulation generalized to the PAW method[26], we obtained the gross population $Z_i(t)$ for the ith atom and the bond-overlap population $O_{ij}(t)$ for a pair of ith and j^{th} atoms as a function of time t. From $Z_i(t)$, we estimate the charge of atoms, and $O_{ij}(t)$ gives a semi-quantitative estimate of the strength of covalent bonding between atoms. As the atomic-basis orbitals, we used numerical pseudo-atomic orbitals, which were obtained for a chosen atomic energy so that the first node occurs at the desired cutoff radius[27]. To increase the efficiency of the expansion, the numerical basis orbitals were augmented with the split-valence method[28]. The resulting charge spillage, which estimates the error in the expansion, was only 0.15 %, indicating the high quality of the basis orbitals. Figure S1 shows the characters of photoexcited electron and hole charge densities during NAQMD simulation, which are projected onto pseudoatomic orbitals of different angular momenta (*i.e.*, s, p, and d) centered around different atoms.

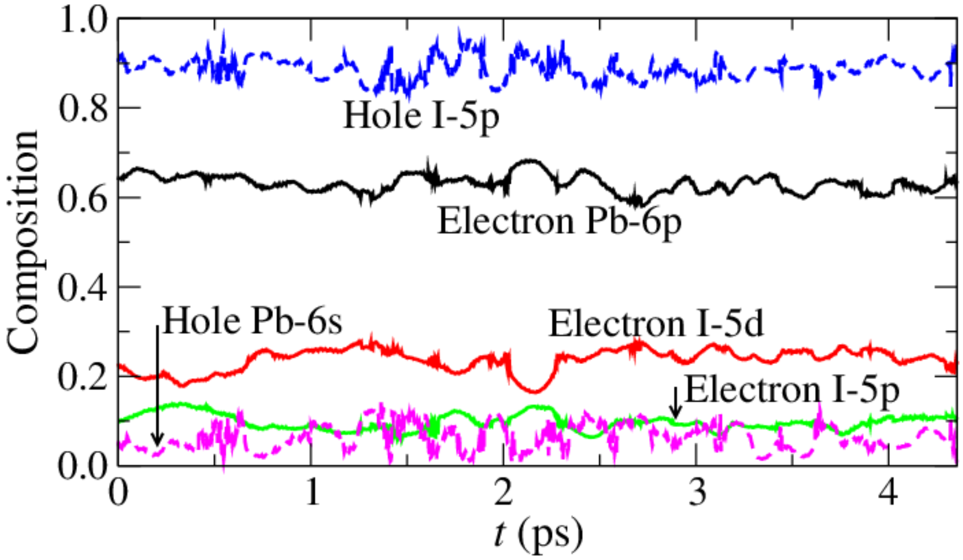

Figure S1. Time evolution of the projection of photoexcited quasielectron and quasihole charge densities onto different angular momenta (*i.e.*, s, p, and d) on different atoms in a 2×2×2 unit-cell NAQMD simulation.

Finite-size effects: In the 2×2×2 system containing 96 atoms, time-averaged electron and hole participation numbers are 7.4 ± 3.1 and 9.8 ± 3.9, respectively. Namely, even in the smaller 2×2×2 system, a charge carrier occupies only 8~10 % of the total volume. The electron and hole wave functions are thus not percolating through the system and act as well defined wave packets, likely due to thermal disorder[29]. In order to quantify the effect of periodic boundary conditions (PBC), we have performed a PBC-compatible calculation of wave function's center of mass (COM). For the *x* direction, for example, it is given by

$$x_{\text{COM-PBC}} = \frac{L}{2\pi} \operatorname{Im} \ln \int d\mathbf{r} \, |\psi(\mathbf{r})|^2 \exp(i \frac{2\pi}{L} x), \tag{S1}$$

where L is the simulation box length in the x direction and $\psi(\mathbf{r})$ is the wave function. Figure S2 compares the hole mean square displacement (MSD) in the 2×2×2 system computed using Eq. (S1) with the original MSD calculation in the main text. Both calculations agree within 14% of the error bar of the new MSD calculation, demonstrating that finite-size effects are negligible.

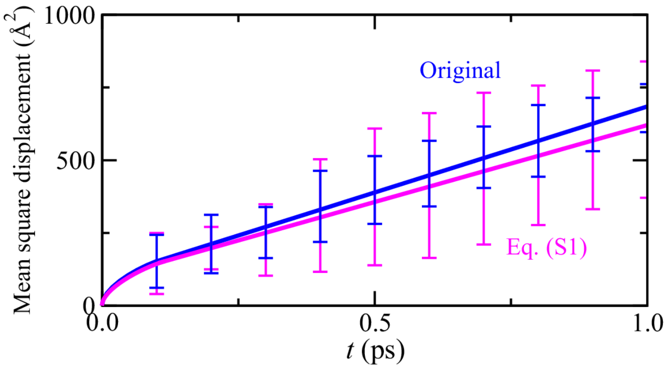

Figure S2. Hole mean square displacement in the 2×2×2 system. The original calculation (blue) is compared with that based on Eq. (S1) (magenta).

Radiative recombination time: We calculated the spontaneous-emission contribution to the radiative recombination time from the oscillator strength as in Refs. 30,31, where we used the refractive index of 2.61[32].

Electric polarizability: We computed the dielectric constant ε using the fluctuation-dissipation theorem[33,34]:

$$\varepsilon = 1 + \frac{4\pi}{3k_B TV}\left(\langle \mathbf{M}^2 \rangle - \langle \mathbf{M} \rangle^2\right), \tag{S2}$$

where k_B is the Boltzmann constant, T is temperature, V is the volume of the simulation box, and $\mathbf{M}$ is the total dipole moment. $\langle\rangle$ denotes time average.

Movies of Carrier Transport.

QuickTime movie, S1.mov, animates isosurfaces of the quasielectron (red) and quasihole (blue) charge densities in a 3×3×3 unit-cell NAQMD simulation, where the threshold charge density is 1×10^{-4} a.u.$^{-3}$. In the movie, H, C, N, I and Pb atoms are shown as white, cyan, blue, green and brown spheres, respectively. QuickTime movie, S2.mov, shows quasielectron and quasihole center-of-mass positions as yellow and black spheres, respectively, as well as the atoms occupied by quasielectron and quasihole charge densities, respectively, as red and blue transparent spheres.

Supplementary References.

1 Blochl, P. E. Projector augmented-wave method. *Phys Rev B* **50**, 17953-17979 (1994).

2 Hohenberg, P. & Kohn, W. Inhomogeneous electron gas. *Phys Rev* **136**, B864-B871 (1964).

3 Perdew, J. P., Burke, K. & Ernzerhof, M. Generalized gradient approximation made simple. *Phys Rev Lett* **77**, 3865-3868 (1996).

4 Louie, S. G., Froyen, S. & Cohen, M. L. Non-linear ionic pseudopotentials in spin-density-functional calculations. *Phys Rev B* **26**, 1738-1742 (1982).

5 Payne, M. C., Teter, M. P., Allan, D. C., Arias, T. A. & Joannopoulos, J. D. Iterative minimization techniques for *ab initio* total-energy calculations - molecular-dynamics and conjugate gradients. *Rev Mod Phys* **64**, 1045-1097 (1992).

6 Kresse, G. & Hafner, J. *Ab-initio* molecular-dynamics simulation of the liquid-metal amorphous-semiconductor transition in germanium. *Phys Rev B* **49**, 14251-14269 (1994).

7 Shimojo, F., Kalia, R. K., Nakano, A. & Vashishta, P. Linear-scaling density-functional-theory calculations of electronic structure based on real-space grids: design, analysis, and scalability test of parallel algorithms. *Comput Phys Commun* **140**, 303-314 (2001).

8 Shimamura, K., Shimojo, F., Kalia, R. K., Nakano, A. & Vashishta, P. Bonding and structure of ceramic/ceramic interfaces. *Phys Rev Lett* **111**, 066103 (2013).

9 Shimojo, F., Ohmura, S., Kalia, R. K., Nakano, A. & Vashishta, P. Molecular dynamics simulations of rapid hydrogen production from water using aluminum clusters as catalyzers. *Phys Rev Lett* **104**, 126102 (2010).

10 Shimamura, K. *et al.* Hydrogen-on-demand using metallic alloy nanoparticles in water. *Nano Lett* **14**, 4090-4096 (2014).

11 Craig, C. F., Duncan, W. R. & Prezhdo, O. V. Trajectory surface hopping in the time-dependent Kohn-Sham approach for electron-nuclear dynamics. *Phys Rev Lett* **95**, 163001 (2005).

12 Hu, C. P., Hirai, H. & Sugino, O. Nonadiabatic couplings from time-dependent density functional theory: formulation in the Casida formalism and practical scheme within modified linear response. *J Chem Phys* **127**, 064103 (2007).

13 Tapavicza, E., Tavernelli, I. & Rothlisberger, U. Trajectory surface hopping within linear response time-dependent density-functional theory. *Phys Rev Lett* **98**, 023001 (2007).

14 Zhang, X., Li, Z. & Lu, G. First-principles simulations of exciton diffusion in organic semiconductors. *Phys Rev B* **84**, 235208 (2011).

15 Mou, W., Ohmura, S., Shimojo, F. & Nakano, A. Molecular control of photoexcited charge transfer and recombination at a quaterthiophene/zinc oxide interface. *Appl Phys Lett* **100**, 203306 (2012).

16 Casida, M. E. in *Recent Advances in Density Functional Methods (Part I)* (ed D. P. Chong) pp. 155-192 (World Scientific, 1995).

17 Tully, J. C. Molecular dynamics with electronic transitions. *J Chem Phys* **93**, 1061-1071 (1990).

18 Schmidt, J. R., Parandekar, P. V. & Tully, J. C. Mixed quantum-classical equilibrium: surface hopping. *J Chem Phys* **129**, 044104 (2008).

19 Prezhdo, O. V. Mean field approximation for the stochastic Schrodinger equation. *J Chem Phys* **111**, 8366-8377 (1999).

20 Jasper, A. W., Stechmann, S. N. & Truhlar, D. G. Fewest-switches with time uncertainty: a modified trajectory surface-hopping algorithm with better accuracy for classically forbidden electronic transitions. *J Chem Phys* **116**, 5424-5431 (2002).

21 Jaeger, H. M., Fischer, S. & Prezhdo, O. V. Decoherence-induced surface hopping. *J Chem Phys* **137**, 22A545 (2012).

22 Shimojo, F. *et al.* Large nonadiabatic quantum molecular dynamics simulations on parallel computers. *Comput Phys Commun* **184**, 1-8 (2013).

23 Shimojo, F. *et al.* A divide-conquer-recombine algorithmic paradigm for multiscale materials modeling. *J Chem Phys* **140**, 18A529 (2014).

24 Mulliken, R. S. Electronic population analysis on LCAO-MO molecular wave functions. I. *J Chem Phys* **23**, 1833-1840 (1955).

25 Segall, M. D., Shah, R., Pickard, C. J. & Payne, M. C. Population analysis of plane-wave electronic structure calculations of bulk materials. *Phys Rev B* **54**, 16317-16320 (1996).

26 Shimojo, F., Nakano, A., Kalia, R. K. & Vashishta, P. Electronic processes in fast thermite chemical reactions: a first-principles molecular dynamics study. *Phys Rev E* **77**, 066103 (2008).

27 Sankey, O. F. & Niklewski, D. J. *Ab initio* multicenter tight-binding model for molecular-dynamics simulations and other applications in covalent systems. *Phys Rev B* **40**, 3979-3995 (1989).

28 Soler, J. M. *et al.* The SIESTA method for *ab initio* order-*N* materials simulation. *J Phys Condens Matter* **14**, 2745-2779 (2002).

29 Nakano, A., Vashishta, P. & Kalia, R. K. Electron transport in disordered systems - a nonequilibrium quantum-molecular-dynamics approach. *Phys Rev B* **43**, 10928-10932 (1991).

30 Shimamura, K., Yuan, Z., Shimojo, F. & Nakano, A. Effects of twins on the electronic properties of GaAs. *Appl Phys Lett* **103**, 022105 (2013).

31 Yuan, Z. & Nakano, A. Self-replicating twins in nanowires. *Nano Lett* **13**, 4925-4930 (2013).

32 Loper, P. *et al.* Complex refractive index spectra of $CH_3NH_3PbI_3$ perovskite thin films determined by spectroscopic ellipsometry and spectrophotometry. *J Phys Chem Lett* **6**, 66-71 (2015).

33 Ogata, S. *et al.* Variable-charge interatomic potentials for molecular-dynamics simulations of TiO_2. *J Appl Phys* **86**, 3036-3041 (1999).

34 Sharma, M., Resta, R. & Car, R. Dipolar correlations and the dielectric permittivity of water. *Phys Rev Lett* **98**, 247401 (2007).
